# Haloarchaeal Carotenoids: Healthy Novel Compounds from Extreme Environments

**DOI:** 10.3390/md17090524

**Published:** 2019-09-06

**Authors:** Micaela Giani, Inés Garbayo, Carlos Vílchez, Rosa María Martínez-Espinosa

**Affiliations:** 1 Biochemistry and Molecular Biology Division, Agrochemistry and Biochemistry Department, Faculty of Sciences, University of Alicante, Ap. 99, E-03080 Alicante, Spain; 2 Algal Biotechnology Group, University of Huelva and Marine International Campus of Excellence (CEIMAR), CIDERTA and Faculty of Sciences, 21071 Huelva, Spain (I.G.) (C.V.)

**Keywords:** haloarchaea, isoprenoid, carotenoids, bacterioruberin, natural biosources, microbial blooms, antioxidant

## Abstract

Haloarchaea are halophilic microorganisms belonging to the archaea domain that inhabit salty environments (mainly soils and water) all over the world. Most of the genera included in this group can produce carotenoids at significant concentrations (even wild-type strains). The major carotenoid produced by the cells is bacterioruberin (and its derivatives), which is only produced by this kind of microbes and few bacteria, like *Micrococcus roseus*. Nevertheless, the understanding of carotenoid metabolism in haloarchaea, its regulation, and the roles of carotenoid derivatives in this group of extreme microorganisms remains mostly unrevealed. Besides, potential biotechnological uses of haloarchaeal pigments are poorly explored. This work summarises what it has been described so far about carotenoids from haloarchaea and their production at mid- and large-scale, paying special attention to the most recent findings on the potential uses of haloarchaeal pigments in biomedicine.

## 1. Haloarchaea

Hypersaline environments represented by hypersaline lakes, soils, springs, solar salterns, and rock salt deposits are widely distributed. These environments present higher salinities than sea water (approximately between 20% and 35% (*w*/*v*)). Organisms characterised by their high salt tolerance/requirements inhabit these ecosystems [1]. The organisms living under these conditions are usually termed “Halotolerants/Halophiles” since their growth range extends above 2.5 M of total salt concentration.

Halophilic microorganisms can be found in bacteria and archaea domains. However, microorganisms requiring high salt concentrations for optimal growth are mainly archaea, grouped into the families *Halobacteriaceae* and *Haloferacaceae*, phylum Euryarchaeota, and archaea domain [2]. These halophilic archaea are widely distributed in salty environments such as marshes, salty ponds or salt lakes, constituting the main microbial populations in such ecosystems [3,4,5,6].

Halophilic archaea are mostly aerobic, although some species can grow anaerobically using nitrate as final electron acceptor (denitrification) [7]. Most of the species are generally red-pigmented. To be alive under these extreme conditions (low water availability and high ionic strength) halophilic microbes have adopted different metabolic adaptations [8]:

(i) Amino acidic residues predominate in halophilic protein surfaces;
(ii) Cells accumulate high KCl intracellular concentrations to deal with the high ionic strength or some osmolytes such as 2-sulfotrehalose [9];
(iii) Cellular bilayers have different composition and structure [10];
(iv) Cells produce an extracellular polymeric substance (EPS) of a protective nature which form a layer surrounding cells, thus providing an effective protection against high salinity [11].

Due to these adaptations, haloarchaea have become a promising and innovative renewable source of different molecules of high interest in biotechnology such as enzymes able to be active at high temperature and high ionic strength [12,13], poly(3-hydroxybutyrate) (PHB) and polyhydroxyalkanoate (PHA) [14,15,16,17], and carotenoids [18,19,20]. Besides, new roles for haloarchaea in wastewater bioremediation processes have also been reported [7,21,22].

## 2. Haloarchaea-Based Biotechnology

Currently, biotechnology has great significance in many fields of application, both industrial and on daily life. The applied use of a broad range of biomolecules such as enzymes as biocatalysts, antioxidants, antibiotics, and bioplastics, among other marketed or investigated natural compounds, is well established and has been the subject of numerous texts and revisions. All halophilic microbes, particularly haloarchaea, show specific metabolic pathways adapted to extreme conditions. Because of that, they are considered as natural sources from which natural biocompounds can be isolated. In addition, their adapted metabolisms to extreme conditions make them potentially suitable for being produced at a large scale with a reduced risk of microbial contamination, which is discussed further in this manuscript. Consequently, and with increasing intensity, there are functions that apply, or intend to, archaea-derived materials.

Halophilic archaea offer an array of actual or potential biotechnological applications [23]. For example, the extremely stable lipids of membranes of these organisms represent a novel drug delivery system [5,24,25,26]. Moreover, the bipolar structure of archaeal lipids offers opportunities for protein-lipid interactions [27] and liposomes with thermostability can be obtained by using archaeal lipids [28].

Self-assembling components from archaea such as the S-layer glycoprotein and bacterioopsin are of interest due to their nanotechnological potential [29,30]. Polysaccharides secreted from haloarchaea could find use in the oil industry [31], while polymers also secreted from haloarchaea have been tested as a raw material for producing biodegradable plastics [32].

However, several technical difficulties have so far avoided large-scale industrial applications from archaeal cultures; for instance, fermenters must be resistant to corrosion by the media required for the growth of halophiles. In this sense, two extreme halophilic archaea that produce poly-γ-glutamic acid and poly-β-hydroxybutyric acid, respectively, have been cultivated in a bioreactor composed of anticorrosion materials and the accumulation of poly-β-hydroxybutyric acid comprised up to 53% of the dry biomass [33].

Halophilic archaea have also been evaluated for bioremediation, in the treatment of wastewaters in the textile industry, for the degradation of organic pollutants [34] to accelerate remediation of oil-polluted saline environments [35], to promote the removal of heavy metals [36], and for the removal of nitrogenous compounds and oxychlorides from brines [7,21,37].

Finally, halophilic enzymes can catalyse their respective reactions in non-aqueous environments, in water/solvent mixtures, at extremely high pressures, at acid and alkali pH, and at temperatures up to 140 °C, or near the freezing point of water [38,39].

## 3. Carotenoids from Haloarchaea

### 3.1. Types, Characterisation, and Biological Roles

Bibliography about carotenoids of extremophile microorganisms is scarce if compared with all information available about carotenoids from non-extremophile organisms. Thus, little has been written about carotenoid production by archaea and haloarchaea [40]. At the end of the 1960s a few studies on carotenoid production from haloarchaea were reported [41,42]. At that time, just a simple characterisation of the carotenoids was done, mainly involving the purification of the pigments by column and thin-layer chromatography, quantification and characterisation by their visible, ultraviolet, infrared, proton magnetic resonance, and mass spectra, and monitorization of the spectra of their acetyl or silyl derivatives and/or dehydrated products [41,43].

From that date up to now, it has been demonstrated that C_50_ carotenoids as bacterioruberin (which is usually the main carotenoid from halophilic archaea) and its precursors (2-isopentenyl-3,4-dehydrorhodopin (IDR), bis-anhydrobacterioruberin (BABR), and mono-anhydrobacterioruberin (MABR)) are synthetised by most members of the haloarchaea group [18,41,43,44].

Other carotenoids such as β-carotene, lycopene, and phytoene are also produced by these species but at lower or very low concentrations as it happens with lycopersene, cis- and trans-phytoene, cis- and trans-phytofluene, neo-β-carotene, and neo-α-carotene [45]. Given their smaller presence, they are probably precursors for the synthesis of other carotenoids including lycopene, retinal, and the members of the bacterioruberin group [46].

The most widely used analytical method to identify and quantify carotenoids by halophilic archaea is spectrophotometry after separation, or not, by thin-layer chromatography or high-performance liquid chromatography (HPLC). But there are some limitations to the latter carotenoid identification procedure that the coupling of HPLC with mass spectrometry can solve providing identification based on their molecular mass and their fragmentation with high sensitivity and selectivity [47,48]. Nuclear magnetic resonance combined to HPLC can help with isomers structure resolution [49]. Besides, Raman spectroscopy has been used recently to identify common and less common carotenoids (α-bacterioruberin, salinixanthin, and spirilloxanthin derivatives) in model organisms belonging to the genera *Haloferax*, *Haloarcula*, and *Halobacterium* among others [50] and moreover it can be used to quantify carotenoids with a minimal volume of the sample. Deeper research into techniques to identify carotenoids with high selectivity and sensitivity is required [44,51].

Carotenogenesis and its regulation, as well as the pathways related to the assimilation or degradation of carotenoids in haloarchaea are still unknown [52]. The first studies on these topics were described in the later 1970s; in brief, the synthesis of C_40_ carotenes in *Halobacterium* was described as follows—isopentenyl pyrophosphate (IPP) leads to trans-phytoene, leads to trans-phytofluene, leads to ζ-carotene, leads to neurosporene, leads to lycopene, leads to gamma-carotene, and finally leads to β-carotene. The main difference between this pathway and the one in higher plants is that the cis isomers of phytoene and phytofluene are not on the main pathway of carotene biosynthesis, as they are in plants [53]. Some other studies stated that the addition of C_5_ isoprene units to each end of the lycopene chain could be the main pathway for bacterioruberin synthesis [43,54]. However, more recent studies suggest that more than one biosynthetic pathway could be acting in haloarchaea [55,56]. Some evidence support that lycopene cyclase (OE3983R) converts lycopene to β-carotene in *Halobacterium salinarum* str. NRC-1 [55], and more recently, Dummer *et al*. proposed a simple reaction between lycopene and the bacterioruberin precursor, tetrahydrobisanhydrobacterioruberin, by the lycopene elongase enzyme (*lye*) [57]. In 2015, a biosynthetic pathway of bacterioruberin from lycopene in *Haloarcula japonica* was described. In this case, lycopene is converted to bacterioruberin by incorporating two C_5_ isoprene units, two double bonds, and four hydroxyl groups. However, all associated enzymes and therefore, the complete pathway have not been fully determined yet [58].

Thanks to the “-omics” era and the increasing number of available haloarchaeal genomes several studies have been focused on the metabolic reconstruction and comparative analysis using a few species of the *Halobacterium*, *Haloarcula*, *Haloquadratum,* and *Natronomonas* genera as model organisms [59]. In this context, the biosynthesis of isoprenoids in halophilic archaea was predicted as follows—the isoprenoid precursor IPP is synthesised via the mevalonate pathway. Then, various isoprenoids detected in the membranes of haloarchaea could be synthesised by a series of condensation reactions with IPP, which is added in a head–tail (HT) or head–head (HH) fashion through desaturase reactions ([2H]) [59].

As it can be concluded from the previous section, bacterioruberin and its derivatives (Table 1) are the main carotenoids responsible for the colour of the red archaea of the families *Halobacteriaceae* and *Haloferacaceae* (members of the haloarchaea group). This pigment is in the cell membrane and has a rather different molecular structure compared to the main carotenoids already described from plants, algae, yeast, and fungi. It has a primary conjugated isoprenoid chain length of 13 C = C units with no subsidiary conjugation arising from terminal groups, which only contain four –OH group functionalities [60,61]. Osmotic stress [20,62], compounds as aniline [63], low oxygen tension, high light intensity [64,65], and pH values above the optimal one for growth [20] are in general, factors that induce its synthesis.

Bacterioruberin presents an important biological role as an antioxidant that protects cells against oxidative damage. This antioxidant activity is related to the number of pairs of conjugated double bonds, the length of the carbon chain, and the concentration [66,67]. It contains 13 pairs of conjugated double bonds versus the nine pairs of conjugated double bonds of the β-carotene, which makes bacterioruberin a better radical scavenger than β-carotene [68,69]. Therefore, haloarchaea is resistant to strong light, gamma irradiation, DNA damage resulting from radiography, UV irradiation, and H_2_O_2_ exposure [70,71].

Bacterioruberin increases membrane rigidity acting as a “rivet” in the membrane cells because of its 4-hydroxyl substitutes in the structure. It also decreases water permeability acting as a barrier and allows permeability to oxygen and other molecules, which makes strains able to survive at low temperatures or hypersaline conditions [72,73].

The other biological role of bacterioruberin is being part of rhodopsin complexes. Crystallographic studies have demonstrated that bacterioruberin provides structural support to archaerhodopsin. Archaerhodopsin is a retinal protein-carotenoid complex found in the claret membrane of *Halorubrum* sp. as well as in other species and whose main purpose is energy production [74,75,76,77].

### 3.2. Haloarchaea as Factories to Produce Carotenoids

Several microorganisms have been proposed as renewable, efficient factories for carotenoid production, microalgae being the most widely studied in that respect [78]. However, little attention has been paid to the potential of haloarchaea as carotenoid producers despite their ability to synthesise and accumulate both C_40_ and C_50_ carotenoids [18,20,79,80].

Several reasons probably explain the limited efforts paid in the use of haloarchaea for carotenoid production [69]:

(a) C_40_ carotenoids have attracted most of the attention in research and development of carotenoid production technology due to their increasing commercial value and the increasing interest in the use of carotenoid producing microalgae to obtain them. However, C_50_ carotenoids which attain specific valuable chemical properties remain to be exploited. This can be mainly due to the lack of knowledge on carotenogenesis in haloarchaea. However, these microbes are currently good options as biofactories for high level production of carotenoids given that engineering of haloarchaea is now possible thanks to the increasing knowledge of the molecular basis of carotenogenesis, the higher number of haloarchaea genomes available, and the availability of tools for haloarchaea genic manipulation. Genetic engineering of haloarchaea has been for a long time a limitation due to the nature of the membranes and cellular walls as well as the characteristics of DNA and RNA metabolism [81,82,83,84,85,86,87]. Thus, it has been recently described that the metabolic engineered *Haloferax mediterranei* strain produced lycopene at 119.25 ± 0.55 mg per gram of dry cell weight in shake flask fermentation. The obtained yield was superior compared to the lycopene production observed in most of the engineered *Escherichia coli* or yeast even when they were cultivated in pilot scale bioreactors [81].
(b) No reports on the scale up of carotenoid production processes of haloarchaea have been published or are available.
(c) Little information has been published regarding the biomass productivity of standard cultures of haloarchaea species; obtaining high biomass productivity values is a key issue to make a production process of a valuable compound feasible. This fact is directly connected to the lack of C_50_ carotenoid production attempts at a larger scale. Designing and developing bioprocesses for the production of haloarchaea biomass at pilot scale remains a challenge.
(d) Though the biosynthetic pathway of bacterioruberin has been partially described in some species, deeper knowledge on the regulation of the key metabolic steps of the pathway should still be obtained. In addition, deeper knowledge on the influence of the physical, chemical, and nutritional parameters on the haloarchaeal growth and on the biosynthesis and accumulation of bacterioruberin should enable performing efficient processes of biomass production and pigment accumulation. Only few works have described the effect of parameters like pH, temperature, UV radiation, salt concentrations, and nutrients availability on the carotenogenesis in haloarchaea [20,62,72].

Consequently, the still scarce scientific information on biomass production and carotenoid accumulation by haloarchaeal species is an opportunity to study and determine metabolic, physiological, physical, and chemical conditions that might result in efficient production processes of carotenoid-enriched haloarchaeal biomass [51].

In addition to it, looking at the unique features occurring in the carotenoid producing haloarchaea species, the potentiality of these microorganisms emerges. For instance, haloarchaea species grow at high salt concentrations, this becomes an advantage to avoid or limit bacterial growth other than the target archaeal species [88]. Furthermore, this is a competitive advantage for outdoor production if compared to production of non-halotolerant microorganisms, for instance widespread mesophilic microalgae should probably be used for biotechnological purposes. The presence of salt is always problematic for many elements of the cultivation system, but a suitable salt concentration can be determined such that it enables growth and limits technical problems to the cultivation system derived from excess salt.

One of the advantages of haloarchaea for the production of C_50_ carotenoids is that their biosynthesis can be easily enhanced by transferring the cells from a culture medium of high salt concentration that favours growth (20–25% *w*/*v*) to a culture medium with a lower salt concentration (normally below 16% *w*/*v*) that favours rapid accumulation of bacterioruberin [20,62,89]. That means that C_50_ carotenoid accumulation and the fast cell growth of haloarchaea are not compatible processes. Therefore, the feasible production of carotenoids from haloarchaea should preferably be performed through a 2-stage process consisting of biomass production under high salt concentrations (first) followed by fast carotenoid biosynthesis and accumulation enhancement under low salt concentrations (second). Even taking into account that the final salt concentration used in those processes could be lower than initially expected (around 16% *w*/*v*), one disadvantage worth mentioning regarding the salt concentration used is that it may cause corrosion of the fermenter, and thus, a corrosion-resistant fermenter is necessary. This is one of the main challenges to be addressed next in the future.

Once pigments accumulate inside the haloarchaeal cells, the following step to complete the production process is extraction from the biomass. When carotenoid production is carried out from microalgal cells, well known carotenoid natural producers, extraction can become a key step in terms of process costs. Cells of many microalgal species are difficult to break due to a cell wall composition that is highly resistant to standard cell breaking tools, including the freezing-unfreezing of algal pellets in liquid nitrogen or the use of sonication, among others. One of the key advantages of haloarchaeal species for carotenoid extraction is that low salt concentrations induce cell lysis, which therefore avoids cost investments in terms of energy required to enable efficient cell breaking [90] and make carotenoids easily available for solvent-mediated extraction compared to direct extraction from not broken cells. This means that haloarchaeal cells might be suitable for maximising pigment recovery eventually at lower costs compared to other microorganisms.

Among the factors that have been reported to influence the accumulation of carotenoids in halophilic archaea, pH, temperature, oxygen concentration, light irradiance, and salt concentration are included [20,43,62,65,90]. But on the top of the influence of the referred parameters on the accumulation rate of carotenoids, the first condition that is required to make the process economically feasible is achieving high biomass productivities in the cultures of the haloarchaeal cultures. The few data available about the biomass productivity of haloarchaeal cultures were obtained at laboratory scale and suggest biomass productivity values of about 0.08 g L d^−1^ [18]. These values are low if compared to those obtained in microalgal cultures. This in principle can be a disadvantage for the large-scale production of carotenoids by haloarchaeal species. However, far from being taken as an unbeatable obstacle, the efficient massive production of haloarchaeal biomass must be taken as a challenge. In that respect, efforts might be paid to optimise the culture medium composition and reactor system that enable achieving higher biomass productivities at a large scale.

The afore mentioned low biomass productivity still could become economically competitive if a given valuable carotenoid accumulated at sufficiently high concentrations inside the cells, roughly at least above 1.5–2% on dry weight basis, thus partly compensating the increased production costs per biomass unit. Interestingly, the carotenoids of several haloarchaeal species have been reported to accumulate intracellularly up to 20–25 mg g^−1^ (2–2.5% of biomass dry weight) [89]. This compares well to the intracellular concentrations of carotenoids reported for several microalgal species. Moreover, such a level of intracellular accumulation of carotenoids, 2–2.5% on dry weight basis, is even higher than most of data published for carotenoid accumulation of microalgae which are normally below 1% on dry weight basis, except for *Dunaliella salina* for β-carotene production [91].

As referred, the potential success of haloarchaeal species for carotenoid production lays in the biomass production improvement. There is still plenty of room for improvement of the cultivation process at a pre-industrial scale as the available production data in the literature comes from laboratory experiences. The use of cheap, raw materials as the source of nutrients, the optimisation of the culture medium composition for large scale production, the improvement of the cultivation systems, the development of production strategies at large scale based on two phases—biomass production (growth phase) and carotenoid accumulation (stress phase)—and the development of extraction technology coupled to the cell lysis phase are all key factors to approach a feasible carotenoid production process by haloarchaeal species [72]. In this sense, the development of carotenoid production processes specifically designed for haloarchaea could take advantage from the technical advancements achieved in two last decades in carotenoid production of microalgae, where process optimized stages [92] can seed the path for establishing feasible carotenoid production processes in haloarchaea.

## 4. Recent Applications of Haloarchaeal Carotenoids in Biotechnology and Biomedicine

Despite the obvious sustainable advantages of haloarchaea as factories to produce carotenoids as well the promising properties of haloarchaeal pigments, there is still poor knowledge of its potential uses such as for pigments in biomedicine, biotechnology, pharmacy or even cosmetics.

The major antioxidant effect of haloarchaeal carotenoids has generated biotechnology and biomedicine research interests (Table 2). The main haloarchaeal carotenoid bacterioruberin has been proven to present a higher antioxidant ability when compared to other commercially available carotenoids, such as beta-carotene [69]. Recent studies have shown possible applications of these haloarchaeal carotenoids in the biomedical field. Human hepatoma HepG2 cells treated with sub-lethal concentrations of *Halobacterium halobium* carotenoid extract (0.2, 0.5 and 1.5 μM) led to a significant decrease in cell viability in time and dose-dependent ways [93]. More recently, a dose-dependent antiproliferative effect was also observed in HepG2 cells treated with carotenoid extracts, in this case from *Haloplanus vescus* (62.5 nM^−1^ μM) and *Halogeometricum limi* (about 1 μM) [94]. Haloarchaeal carotenoid extracts have also profitable properties for the treatment of solid tumour reduction in combined therapy with radiation [95].

Carotenoid extract from several haloarchaeal strains showed interesting scavenging activities in a dose-dependent way. The highest activities were those from *Haloferax volcanii*, *Halogranum rubrum*, *Halopelagius inordinatus,* and *Halogeometricum rufum*. Their activities were significantly higher (*p* < 0.05) when compared with β-carotene activity. Therefore, Hou and Cui suggested that haloarchaeal carotenoids might be stronger radical scavengers than β-carotene [94]. Also, carotenoid extracts from haloarchaea have protected cells exposed to oxidative stress by arachidonic acid and H_2_O_2_, separately. Thus, carotenoid-treated cells showed an increase in viability as compared to non-treated cells [93]. A few years ago, this protective effect was confirmed in erythrocytes exposed to an H_2_O_2_ mediated haemolysis, being again more effective than β-carotene with a 3.9–6.3 fold-change [94].

Besides that, Zalazar et al. 2019 tested whether bacterioruberin extracts from a genetically modified *Haloferax volcanii* could reverse the damage caused by freezing and thawing to ram sperm cells. Bacterioruberin extracts exerted beneficial effects on the viability of sperm cells by reducing the apoptotic and necrotic population, as well as improving the sperm motility possibly by stabilising cell membranes after thawing [96].

On the other hand, bacterioruberin extracts from halophilic bacteria showed significant antimicrobial activity against a wide spectrum of microorganisms including bacteria and fungi, suggesting an interesting biomedical application [97]. Nevertheless, this antimicrobial activity has not been evaluated yet in bacterioruberin extracts from haloarchaea and would be a very interesting approach given their properties.

Regarding the biotechnology field, bacterial and archaeal pigments are a promising alternative to synthetic pigments given their higher biodegradability and less environmental-involved risks; while also offering a huge potential to several applications. It is well known that carotenoids can be used as colorants in food products and cosmetics, as well as feed additives for poultry and crustaceans among others [98]. A recent study has proposed all-trans-bacterioruberin from bacteria as a UV filter because of the absence of phototoxic activity [99]. However, provided the complex structure of the skin and the high molecular weight of these carotenoids (500 Da), there are some concerns about the possible low skin penetration, and therefore, the low in vivo efficacy [100]. For this reason, studies regarding the effectiveness of these pigments as SPF should be developed to prove if indeed these compounds might be used as UV filters or as adjuvants for cosmetics formula. Haloarchaeal carotenoids have not been evaluated yet with this purpose. Another biotechnological application of haloarchaeal extracts is their use as protective agents against radiation skin damage since they show a wide range of impact on the rehabilitation of skin tissue [101].

Although further studies are still required to get the full benefit out of it, all recent approaches point to the interesting applicability of haloarchaeal carotenoids in biomedical and biotechnology fields.

## 5. Conclusions

Several studies demonstrated that some haloarchaeal species (wild-type strains) produce significant concentrations of carotenoids, which are highly marked demanding. Thus, haloarchaea constitute a promising biosource for carotenoid production whose production at large scale by means of suitable bioprocess engineering tools, mainly specifically designed bioreactors, is still challenging.

The main reasons that make haloarchaea suitable for carotenoid production are—(i) many haloarchaeal species possess high carotenoid production availability; (ii) haloarchaeal can grow easily using suitable bioprocess engineering tools (bioreactor); (iii) downstream processes related to carotenoid isolation from haloarchaea are relatively quick, easy, and cheap; (iv) carotenoid production by haloarchaea can be improved by genetic modification or even by modifying several cultivation aspects such as nutrition, growth pH, or temperature; (v) carotenoids are needed to support plant and animal life and human well-being; and (vi) carotenoids are compounds highly demanded by pharmaceutical, cosmetic, and food markets.

At the time of writing this work, the number of studies on the potential benefits of the carotenoids produced by haloarchaea on human health is still scarce. Thus, more efforts should be made to address not only this question, but also other open marks related to carotenoid synthesis and degradation in haloarchaea; such analysis would lead to a better understanding of the spatial distribution and function of different carotenoids and their derivatives in response to environmental and developmental signals. This knowledge may facilitate further progress in the field of carotenoid metabolic engineering in haloarchaea and it would contribute to evaluate whether haloarchaea are good sources for carotenoid production at a large scale.

## Figures and Tables

**Table 1:** Structures, common and scientific names of bacterioruberin, and its most abundant derivatives.

Common Name	Scientific Name	Molecular Formula	Chemical Structure (Stereoisomers)
Bacterioruberin	2,2′- bis(3- hydroxy- 3- methylbutyl)- 3,4,3′,4′- tetradehydro- 1,2,1′,2′- tetrahydro- γ,γ- carotene- 1,1′- diol	C_50_H_76_O_4_	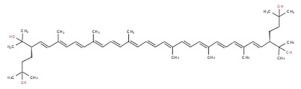 (2*S*,2′*S*)- 2,2′- bis(3- hydroxy- 3- methylbutyl)- 3,4,3′,4′- tetradehydro- 1,2,1′,2′- tetrahydro- γ,γ- carotene- 1,1′- diol
Monoanhydrobacterioruberin	30- (2- hydroxypropan- 2- yl)- 2,6,10,14,19,23,27,33- octamethyl- 3- (3- methylbut- 2- en- 1- yl)tetratriaconta- 4,6,8,10,12,14,16,18,20,22,24,26,28- tridecaene- 2,33- diol	C_50_H_74_O_3_	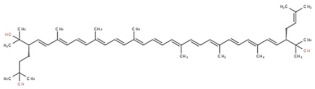 (3*S*,4*E*,6*E*,8*E*,10*E*,12*E*,14*E*,16*E*,18*E*,20*E*,22*E*,24*E*,26*E*,28*E*,30*S*)- 30- (2- hydroxypropan- 2- yl)- 2,6,10,14,19,23,27,33- octamethyl- 3- (3- methylbut- 2- en- 1- yl)tetratriaconta- 4,6,8,10,12,14,16,18,20,22,24,26,28- tridecaene- 2,33- diol
Bisanhydrobacterioruberin	2,6,10,14,19,23,27,31- octamethyl- 3,30- bis(3- methylbut- 2- en- 1- yl)dotriaconta- 4,6,8,10,12,14,16,18,20,22,24,26,28- tridecaene- 2,31- diol	C_50_H_72_O_2_	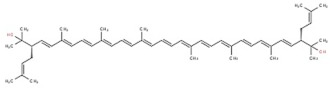 (3*S*,4*E*,6*E*,8*E*,10*E*,12*E*,14*E*,16*E*,18*E*,20*E*,22*E*,24*E*,26*E*,28*E*,30*S*)- 2,6,10,14,19,23,27,31- octamethyl- 3,30- bis(3- methylbut- 2- en- 1- yl)dotriaconta- 4,6,8,10,12,14,16,18,20,22,24,26,28- tridecaene- 2,31- diol

**Table 2:** Recent applications of haloarchaeal carotenoids in biotechnology and biomedicine.

Carotenoid Origin	Biomedical Application	Reference
*Halobacterium halobium, Haloplanus vescus* and *Halogeometricum limi* carotenoid extracts	Decrease in cell viability in human hepatoma HepG2 cells	[93,94]
Haloarchaeal carotenoid extracts	Treatment of solid tumour reduction in combined therapy with radiation	[95]
*Haloferax volcanii*, *Halogranum rubrum*, *Halopelagius inordinatus* and *Halogeometricum rufum* extracts	Scavenging activity	[94]
*Haloarchaeal* extracts	Protection against oxidative stress by arachidonic acid and H_2_O_2_	[93]
*Haloarchaeal* extracts	Protection against H_2_O_2_ in erythrocytes	[94]
*Haloferax volcanii* bacterioruberin extract	Beneficial effects on the viability of sperm cells	[96]
*Halophilic bacterial extracts*	Antimicrobial activity	[97]
